# Stem Cell-Derived Models of Neural Crest Are Essential to Understand Melanoma Progression and Therapy Resistance

**DOI:** 10.3389/fnmol.2019.00111

**Published:** 2019-05-01

**Authors:** Lionel Larribère, Jochen Utikal

**Affiliations:** ^1^Skin Cancer Unit, German Cancer Research Center (DKFZ), Heidelberg, Germany; ^2^Department of Dermatology, Venereology and Allergology, University Medical Center Mannheim, Ruprecht-Karl University of Heidelberg, Mannheim, Germany

**Keywords:** neural crest, melanoma, iPSCs, stem cells, resistance

## Abstract

During development, neural crest (NC) cells are early precursors of several lineages including melanocytes. Along their differentiation from multipotent cells to mature melanocytes, NC cells will go through successive steps which require either proliferative or motile capacities. For example, they will undergo Epithelial to Mesenchymal Transition (EMT) in order the separate from the neural tube and migrate to their final location in the epidermis (Larribere and Utikal, 2013; Skrypek et al., 2017). The differentiated melanocytes are the cells of origin of melanoma tumors which progress through several stages such as radial growth phase, vertical growth phase, metastasis formation, and often resistance to current therapies. Interestingly, depending on the stage of the disease, melanoma tumor cells share phenotypes with NC cells (proliferative, motile, EMT). These phenotypes are tightly controlled by specific signaling pathways and transcription factors (TFs) which tend to be reactivated during the onset of melanoma. In this review, we summarize first the main TFs which control these common phenotypes. Then, we focus on the existing strategies used to generate human NCs. Finally we discuss how identification and regulation of NC-associated genes provide an additional approach to improving current melanoma targeted therapies.

## Introduction

Human embryonic pluripotent stem cells (hESCs) were first isolated in 1998 (Thomson et al., 1998). Derived from the inner mass of a human blastocyst, hESCs have the property to differentiate into all tissues of the human body, except embryonic annexes. This revolutionary discovery allowed researchers to investigate for the first time the human biology at its very early stages on one hand, and to plan stem cell-based therapeutic strategies on the other hand. The utilization of embryonic material to generate hESC lines is nevertheless inherently controversial due to ethical reasons. More recently, new innovations in induced pluripotent stem cells (hiPSCs) eschewed the need for embryonic tissue entirely, via transcription factor (TF)-based reprogramming of somatic cells (Takahashi et al., 2007). During development, neural crest (NC) cells are transient multipotent cells which derive from the dorsal part of the neural tube. This multipotency leads to the differentiation of a wide range of tissues including peripheral nerves, cornea, cartilage, bone, teeth, cardiac cells, connective tissues, and melanocytes. Most of our current knowledge on NC differentiation has been provided by mice or rats models, and from vertebrates such as chicken or quail. Therefore, the hiPSCs approach is particularly attractive for the generation of high amount of human material. In addition, it allows the establishment of patient-specific *in vitro* models for NC associated diseases. Importantly, these models represent valuable alternative of drug testing or cell/gene therapy for diseases with so far no therapeutic options. Moreover, since melanoma is considered as a NC-derived tumor, these PSC-based models bring an additional and powerful tool to investigate the transformation of this tumor entity and its response to the drugs used in the clinic. About half melanoma patients carry a *BRAF* mutation and are typically given combined BRAF and MEK inhibitors such as dabrafenib and trametinib, vemurafenib and cobimetinib, and encorafenib and binimetinib (FDA-approved) (Cheng et al., 2013; Kugel and Aplin, 2014; Long et al., 2014, 2017; Rizos et al., 2014; Johnson et al., 2015; Dummer et al., 2017). Unfortunately, most of these patients will eventually develop a resistance to these drugs with reactivation of MAPK and PI3K-AKT pathways. In addition, the regulation of the tumor microenvironment and of the immune response at the tumor site may have direct impact on the efficiency of immune checkpoint inhibitors which are often proposed to drug-resistant patients.

The objective of this review is to emphasize to power of stem cell-based *in vitro* models of NCs as a comparative and predictive tool for the study of melanoma progression and resistance to cancer therapies. We will therefore examine TFs, role of which has been described both during the development of human NC cells and during melanoma initiation or progression. Then, we will present several differentiation protocols which are used to generate human NC cells from stem cells. Finally, we will discuss the implications of the key regulation of such TFs during melanoma therapy resistance, and the high pertinence of investigating lineage specific signalings in order to improve our understanding of how melanoma still overcomes current treatments in the clinic.

## Similitude Between Melanocyte Specification and Melanoma Progression

As explained above, melanocytes originally derive from the NC cells which commit to this lineage via the expression of specific TFs in a time-dependent manner. Indeed, SRY (sex determining region Y)-Box 10 (SOX10) and Paired box protein 3 (PAX3) are TFs expressed in the NCs, which play a role in the specification of several NC derivatives and in particular of melanocytes. *SOX10* haploinsufficiency for example, leads to Waardenburg syndrome type IV with ganglionic megacolon due to the loss of ganglion cells, pigmentary abnormalities due to the lack of melanocytes and deafness due to the loss of sensory innervation. Mutations of *PAX3* have been identified in Waardenburg syndrome type I and the related *splotch* mouse model presents white spots due to defects in NCs (Moase and Trasler, 1992; Pingault et al., 1998; Watanabe et al., 1998; Potterf et al., 2000; Verastegui et al., 2000; Hornyak et al., 2001).

Interestingly, SOX10 and PAX3 are described to colocalize at melanocyte-specific regulatory elements in the promoter of microphthalmia-associated transcription factor (MITF) (Seberg et al., 2017). The latter was originally described as the master regulator of melanocyte lineage specification during development and mutations of this gene lead to the Waardenburg Syndrome type II with permanent hearing loss, pigmentation defects of the eyes, the skin and the hair (Read and Newton, 1997; Hallsson et al., 2000).

Additionally, the POU TF family and BRN2 in particular is thought to be important for melanocyte lineage development (Cook and Sturm, 2008). Although many *in vitro* studies could correlate reduced BRN2 expression with melanocyte differentiation, its expression and role *in vivo* seems to be less clear (Colombo et al., 2012).

**FIGURE 1:**
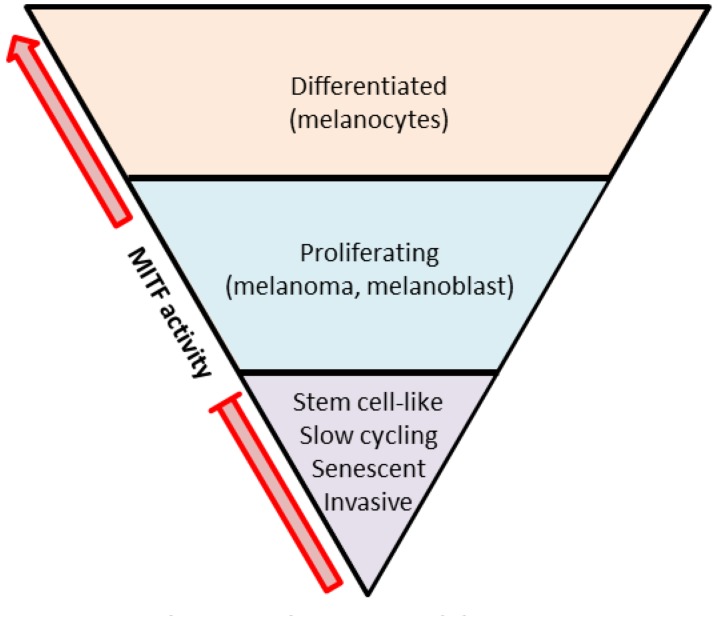
The MITF rheostat model. Low MITF activity is found in slow-cycling, stem cell-like, or invasive cells. Proliferating cells possess intermediate MITF activity. High MITF activity is found in differentiated and less proliferating cells (adapted from Goding, 2011).

It is striking to see that these TF have all been reported to play a key role in the regulation of tumor cells. The level of MITF activity for example, which depends on its expression but also on its post-translational modifications, plays a crucial role in the behavior of melanoma cells. The so-called MITF rheostat model developed by the Goding’s lab proposes that increasing gradient of MITF activity influences cell phenotypes ranging from senescence and invasion to proliferation and differentiation (Figure 1) (Goding, 2011). Thus, on one hand, high-MITF expressing cells will harbor a proliferating phenotype with activation of MITF targets involved in survival and proliferation such as BCL2 and CDK2 (McGill et al., 2002; Du et al., 2004). On the other hand, low-MITF expressing cells will downregulate MITF’s target DIA1 and increase ROCK signaling, leading to an invasive phenotype (Carreira et al., 2006; Hoek et al., 2006; Wellbrock and Arozarena, 2015). Based on this MITF rheostat model, the concept of phenotype switch has emerged a few years later to explain the progression of melanoma. That is, tumor phenotypic heterogeneity would rely on a switch between two mutually exclusive subpopulations of cells. One population with highly pigmented, proliferative population driven by MITF expression and one population with a slow cycling, stem cell-like invasive phenotype with low MITF expression (Hoek et al., 2009; Hoek and Goding, 2010). Of note, the invasive/MITF-low signature is also associated with high expression of the NC-associated gene *FOXD1* which was recently described to play a key role in melanoma migration and invasion via *RAC1b* regulation (Wu et al., 2018).

The expression level and activity of SOX10 was also demonstrated a few years ago, to regulate melanomagenesis and metastases formation in different melanoma mouse models (Shakhova et al., 2012; Cronin et al., 2013). In the first study, it was showed that SOX10 is required for proliferation of melanoma tumor cell and that *SOX10* haploinsufficiency reduces melanoma initiation in the metabotropic glutamate receptor 1 [*Grm1*(Tg)] transgenic mouse model. In the second study, *SOX10* haploinsufficiency in Tyr::Nras^Q61K^INK4a^−/−^ mice counteracted melanoma formation. A study integrating data from transcriptome, open chromatin and histone modification maps of melanoma cultures identified SOX10/MITF as regulator of the proliferative state and AP-1/TEAD as regulator of the invasive state (Verfaillie et al., 2015).

The oncogenic role of PAX3 has also been investigated in many different tumor entities including melanoma, resulting in a potential involvement of this gene in tumor progression (Mascarenhas et al., 2010; Boudjadi et al., 2018).

BRN2 expression inversely correlates with that of MITF in melanoma cells and this relationship is thought to play a key role in the rheostat model mentioned above. As BRN2 represses MITF and drives motility of tumor cells *in vivo*, the BRN2-MITF expression axis may be considered as a driver of melanoma invasion, in addition to TGFβ, JARID1B and β-catenin signalings (Goodall et al., 2008; Pinner et al., 2009; Hoek and Goding, 2010; Roesch et al., 2010). Another member of the POU family, BRN3A, was also studied in the context of the melanocyte lineage. Although its role during development is not yet clear, its expression in melanocytes prevents BRAF-induced senescence and leads to proliferation, suggesting a role for BRN3A in melanoma transformation (Hohenauer et al., 2013; Besch and Berking, 2014).

Together these data bring evidences that TF which are involved in melanocyte specification from NC during development are also tightly regulating the proliferation or invasion phenotype of melanoma cells. It is tempting to suggest that additional unidentified melanocyte lineage-associated TF might exist to completely explain melanoma cells behavior.

## Stem Cell-Based Models of Human Neural Crests

### Neural Crest Derivation From PSCs

The first NC derivation from hESCs or hiPSCs using coculture with bone marrow-derived stromal cells from mice has shown some limitation due to results variability and to the presence of unwanted mouse cells (Lee et al., 2007, 2009, 2012). NC derivation from hESCs or hiPSCs using three-dimentional aggregates called embryoid bodies (EB) has brought to light important mechanisms of NC formation, migration, and differentiation into neurons or melanocytes (Fang et al., 2006; Bajpai et al., 2010; Cimadamore et al., 2012; Rada-Iglesias et al., 2012). Nevertheless, the low efficiency of this protocol remains a hurdle. Nowadays, monoculture differentiation is a preferred method as it does not require feeder cells nor the formation of EBs. Because of the specific NC location at the neural plate border, hPSCs need to be committed to dorsal neuroectoderm in order to efficiently generate NC. Due to the fact that hPSC usually tend to acquire ventral phenotype during neural induction by SMAD inhibition, NC protocols are based on additional inhibition of ALK and activation of WNT signaling with a GSK3β inhibitor (Inman et al., 2002; Cohen and Goedert, 2004; Chambers et al., 2009). In this case, the expression of neural markers PAX6 and SOX2 is downregulated and early NC markers is upregulated (Menendez L. et al., 2011; Chambers et al., 2012; Mica et al., 2013). Moreover, the addition of intermediate concentrations of BMP4, a morphogen known to play a role in NC specification and to act as a mild dorsalizing agent was required for the derivation of NC cells from hPSCs (Jessell, 2000; Reichert et al., 2013; Larribere et al., 2015). Recently, the intermediate BMP4 concentration for efficient NC differentiation was confirmed in a fully defined culture system. Concentrations outside the optimal range would rather favor non-NC lineage differentiation (Hackland et al., 2017).

In order to differentiate NC toward melanocytes, additional endothelin 3 was described (Mica et al., 2013). In this study, NC cells were sorted based on SOX10:GFP expression, and melanoblasts were identified via cKIT expression which is highly expressed in pigmented cells (Ito et al., 1999). The potential involvement of Notch signaling in the NC formation seems to be unclear. One study reported activation of this signaling after WNT agonist-based NC differentiation, however, Notch inhibition was used to increase NC derivation in another similar protocol (Chambers et al., 2012; Noisa et al., 2014). This monoculture approach has also shown some discrepancy, especially regarding the selective markers NGFR and HNK-1. Two populations of cells with NGFR high and NGFR low may coexist during the NC differentiation but only NGFR high population seems to be a marker for NC (Menendez M.I. et al., 2011). Although NGFR expression has been observed in pre- and postmigratory NC cells from human embryos, expression of HNK-1 seems to be questionable (Betters et al., 2010). Therefore, the identification of new human NC markers would greatly improve the current NC differentiation protocols.

### Neural Crest Derivation From Adult Stem Cells

Evidences for the presence of multipotent stem cell populations in adult tissues have been shown. Their differentiation could lead not only to mesenchymal derivatives but also to NC-like cells with label retaining and sphere formation abilities. Moreover, this differentiation could further generate mature melanocytes (Yu et al., 2006; Stevens et al., 2008). Interestingly, adult stem cells expressing NC markers SOX10 and CD271 were also reported to be present in human melanoma (Fang et al., 2005; Civenni et al., 2011).

### Applications for SC-Derived NC

Because of the multipotentiality of the NC, many diseases affecting the NC-derived tissues can be of interest for a SC-based therapy. We will focus here on the melanocyte lineage and its transformed counterpart, the melanoma.

### Melanocyte Lineage

Among the many melanocyte-related diseases, a lot of efforts is being done for albinism and vitiligo. Affecting around 1/20 000 individuals, albinism is a genetic condition affecting the vision and the skin pigmentation leading to higher sensitivity to the sunlight. Patients are therefore more prone to UV-related skin cancer (Federico and Krishnamurthy, 2018). Vitiligo is an acquired chronic hyperpigmentation disorder, which affects less than 1% of the population. In this condition, not only pigment cells are defect but also immune and inflammation responses are deregulated (Passeron, 2017). Melanocyte transplantation for vitiligo has been tested for a long time with mixed results. More recently, new strategies based on the melanocyte stem cells response to UV light have been proposed, to identify the mechanisms of repigmentation (Goldstein et al., 2018). Nevertheless, no SC-based models have been developed so far.

Until the achievement of hiPSCs, the main hurdle in melanocyte research was the limited amount of material one can expect from donor biopsies. Therefore, the virtually unlimited number of melanocytes, which could be generated from either wild type or mutated PSCs, would greatly improve the possibilities of cell therapy, high content pharmaceutical screenings or *in vitro* models of pigmentary disease.

In the mouse system, melanocyte generation from embryonic NCSC has been performed by addition of SCF and END3 in the culture medium (Shakhova and Sommer, 2015). In the human system, protocols of melanocyte differentiation from PSCs through a NC stage have also been described. Efficiency was evaluated by melanosome formation (EM), gene expression analysis, and melanin production under stimulation in a three-dimensional skin reconstruction model (Nissan et al., 2011; Ohta et al., 2011; Mica et al., 2013). The reproducibility of these models will allow to develop genetic disease models with the use of appropriate mutated PSCs. By comparison with non-mutated PSC-derived melanocytes (preferentially isogenic controls via gene editing), this will allow to investigate the molecular mechanisms involved in the disease of interest. Additionally, with the standardization of cellular assay monitoring the defect in melanin synthesis or melanocyte survival, drug testing should become easier to set up in the close future. Few examples of such *in vitro* model have already been described. A model of hypopigmentation disorder called Hermansky-Pudlak syndrome was developed by Mica et al. In this autosomal-recessive disorder, patients can present oculocutaneous albinism, platelet storage disease, immune deficiency, and pulmonary fibrosis. In particular, the patient’s melanocytes suffer defects in the biogenesis of the melanosomes (Oh et al., 1996; Wei, 2006). Mutated hiPSC-derived melanocytes with aberrant number of melanosomes could be used for high-throuput small compound screening with the aim of reversing the phenotype and identify a candidate drug (Mica et al., 2013). Models of the hyperpigmentation disorder Neurofibromatosis syndrome type 1 (NF1) are also available. Patients clinical manifestations include the development of dermal neurofibroma (originating from skin-derived precursor cells) (Le et al., 2009; Tolar et al., 2011) and the development of *café-au-lait* macules (benign melanocytic lesions) (Friedman and Birch, 1997; Gutmann et al., 1997). Mutated hPSC-derived melanocytes tend to produce more melanin than the controls and will eventually enter an oncogene-induced senescence program. These abnormal cells could be used to identify potential targets for this syndrome which otherwise does not allow very good therapeutic options (Larribere et al., 2015; Larribere and Utikal, 2016).

In sum, these data demonstrate the high value of PSC-derived NC models in order to produce large amounts of human melanocytes. These cells can therefore be used for basic research, drug discovery and ultimately in the clinic for patients with melanocyte-associated diseases. For this purpose, the safety of PSC-derived melanocytes will have to be tightly evaluated before being implanted into patients.

### NC-Derived Tumor: Melanoma

The availability of human PSC-derived NC is therefore a big step for the comparison of all – omics with that of NC-derived tumors. For example, we recently identified candidate genes, expression of which was upregulated in SC-derived NC and in melanoma cells when compared to primary melanocytes. We showed that Forkhead Box superfamily member *FOXD1* is required for melanoma progression since its silencing could significantly impair melanoma migration and invasion (Wu et al., 2018). Of note, *FOXD1* can also promote drug resistance of breast cancer (Zhao et al., 2015). We also observed that inhibitor of differentiation 3 (ID3), which belongs to the helix-loop-helix (HLH) TF superfamily, is upregulated in the tumors of melanoma patients after their treatment with a BRAF inhibitor, when compared to before the treatment. Moreover *ID3* silencing leads to an increased melanoma sensitivity to vemurafenib short-term treatment (Sachindra et al., 2017). In addition we could recently correlate the expression of NC-associated genes *GLDC* and *ERRFI1* with melanoma prognosis (Jäger et al., 2019).

These data suggest that melanoma may revert to its NC origins and reinforce the value of SC-derived NC as a melanoma model. Indeed, a zebrafish melanoma model has already demonstrated the emergence of NC identity during tumor initiation (Kaufman et al., 2016).

In addition, SC-derived NC models may also provide high amounts of human melanoblasts when the differentiation is pushed forward in this lineage. We have therefore analyzed the transcriptome of PSC-derived melanoblasts and that of melanoblasts otherwise generated from the dedifferentiation of mature melanocytes (Larribère et al., 2018). Although each cell population presented a specific profile, both expressed melanoblast markers (such as *MITF*, *KIT*, *KIT-L*, *or SNAI2*). Interestingly, when these cells where compared to mature melanocytes in a gene expression analysis, a subset of TFs including *JUN*, *AP2C*, *ID2*, *ID3*, and *STAT1* was enriched, suggesting a specific involvement of these genes during embryonic development. In this study, the transcriptome of melanoma cells which were exposed to short-term (adaptive resistance) or long-term (acquired resistance) treatment with vemurafenib was also included. Strikingly, the melanoblasts presented more similarities with adaptive resistant melanoma than with acquired resistant melanoma which implies that resistance gene regulation may overlap to some extent with that observed during melanoblast differentiation. Nevertheless, this gene regulation may vary between adaptive and acquired resistance due to obvious differences in kinetics. When compared to vemurafenib-sensitive melanoma cells, melanoblasts and both resistant melanoma cells presented gene enrichment in cell cycle regulation, DNA damage regulation, metabolism of amino acids and nucleotides, JAK–STAT and p53 signalings. Moreover, genes regulated in melanoblasts and acquired resistant melanoma cells were specifically involved in antigen presentation (HLA), cellular movement (CDC42 or ACTIN signaling), and ILK signaling. HLA downregulation in particular, was already described in acquired MAPKi-resistant tumors from melanoma patients (Hugo et al., 2015). When grouped with adaptive resistance melanoma cells, each melanoblast population (derived from PSC or melanocytes) showed a specific gene regulation compared to vemurafenib-sensitive melanoama cells. For example, PSC-derived melanoblasts regulated genes were involved in cell cycle control, DNA damage response and glycolysis whereas melanocyte-derived melanoblasts regulated genes were involved in CDK5 signaling or belonging to ID and FOX TF families. Together, these data show the importance of using human SC-derived melanocyte precursors to better understand the mechanisms of melanoma progression and, combined with additional information on genetic or epigenetic changes, will allow to identify new therapeutical targets.

## Key Role of Mitf and Sox10 in Melanoma Patient Therapy Resistance

So far, mechanisms underlying therapy resistance in melanoma are not fully understood and rely at least in part on the heterogeneity of the tumor. *BRAF* amplifications, *BRAF* splice variants and mutations in *MEK1/2* are the main identified sources of MAPK and PI3K pathway reactivation. However, evidence for non-mutational drug tolerance mechanisms in a small fraction of the tumor is raising (Sharma et al., 2010; Su et al., 2017). Indeed the notion of tumor cell subpopulations is yet deeply investigated at the single-cell level and markers such as MITF or SOX10 have been suggested to identify them.

A subpopulation of slow-cycling but highly invasive cells with gene signature containing low levels of MITF and SOX10 but high levels of AXL or EGFR is described to be resistant to MAPK inhibition (Konieczkowski et al., 2014; Müller et al., 2014; Dugo et al., 2015; Verfaillie et al., 2015; Kemper et al., 2016; Shaffer et al., 2017). Similarly, a slow-cycling cell subpopulation with low MITF and high NGFR expression was described to be resistant to short-term RAF inhibition (Fallahi-Sichani et al., 2017; Su et al., 2017). Based on the upregulation of these tyrosine kinase receptors (AXL, EGFR, NGFR), the possibilities of interfering with this particular class of proteins are currently under investigation. Moreover, MITF repression during starvation-induced translational reprogramming has been shown to correlate with drug and immunotherapy resistance of melanoma cells (Falletta et al., 2017). In the context of nutrient starvation in the tumor microenvironment, activation of the translation initiation factor eIF2B leads to both translational inhibition and ATF4-mediated transcriptional downregulation of MITF.

Together, these observations are in line with the hypothesis of a non-genetic phenotype switch from proliferative to invasive state with low MITF expression, in response to therapy.

However, high expression levels of MITF have also been shown to either drive reversible drug resistance or to maintain drug resistance in melanoma (Johannessen et al., 2013; Smith et al., 2013, 2016; Wellbrock and Arozarena, 2015). These apparently conflicting results presenting distinct MITF high and/or MITF low transcriptional states may not be mutually exclusive and their respective contribution to drug resistance *in vivo* is still unknown.

Recently, Rambow et al. (2018) demonstrated in an elegant manner that at least four transcriptional drug-tolerant states coexist within a single tumor after inhibition of RAF and MEK. After single cell RNA sequencing of *BRAF*-mutated patient-derived xenografts which were subjected to combined drug inhibition, the authors identified one cell population with a major regulation of a NC stem cell gene signature driven by the activation of nuclear receptor *RXRG*. Moreover, pharmacological inhibition of RXRG could delay the drug resistance onset of melanoma cells (Rambow et al., 2018). Again, these data confirm the NC conversion of a cell subpopulation within one melanoma tumor.

Low SOX10 expressing melanoma cells have been shown to adapt to BRAF inhibition. The authors found an upregulation of EGFR and a slow-growing phenotype after vemurafenib stimulation. Interestingly, two other tyrosine kinase receptors were regulated in addition to EGFR (PDGFRb and ERBB3). Since all three receptors are common targets of the TGFβ signaling, it suggests an involvement of this pathway in the response to vemurafenib treatment. Although a TGFβ gene signature was associated with the «invasive phenotype» described by Hoek, a link to drug resistance is so far unknown. In addition, the authors observed a reversibility of this resistant state when taking the cells off the drug, a characteristic which could offer an alternative to a subpopulation of *BRAF*-mutated patients who has stopped its treatment (Sun et al., 2014). Repression of *SOX10* expression was also suggested in melanoma cells which were upregulating ID3 after vemurafenib short-term stimulation. However, it was unclear if *SOX10* suppression was a direct or indirect consequence of the treatment (Sachindra et al., 2017).

Recently, the involvement of SOX10 in melanoma drug resistance was reanalyzed at the single cell level. Rare cell populations with non-genetic differences have been identified during the establishment of vemurafenib resistance and the authors proposed the following model: non-resistant tumor cells can switch to a pre-resistant reversible state with expression of AXL, EGFR, and NGFR but variable expression of SOX10. After vemurafenib stimulation, these pre-resistant cells could switch to a resistant state with epigenetically stable changes and a low expression of SOX10. This second switch would be mediated, at least in part, by TFs such as JUN, AP-1, or TEAD (Shaffer et al., 2017). Therefore loss of SOX10 expression in response to BRAF inhibitors leads to a cellular reprogramming involving epigenetic and transcriptomic changes which render the cells slow-growing and drug resistant.

Based on the demonstration that either SOX10 phosphorylation or SOX10 sumoylation play an important role in its expression and in the activation of its downstream targets in melanoma, it would be interesting to investigate the role of SOX10 post-translational modifications in drug resistance (Cronin et al., 2018; Han et al., 2018). Indeed a decrease in the level of Ubiquitin Ligase RNF125 has been described to correlate with SOX10/MITF expression and to promote BRAF inhibitors resistance in melanoma (Kim et al., 2015).

Finally, SOX2, another member of the SOX TF family which regulates stem cells pluripotency in connection with OCT4 and NANOG (Kim et al., 2008) was recently described to regulate anticancer drugs resistance in melanoma (Hüser et al., 2018a,b).

In sum, mechanisms of drug resistance are numerous and distinct subpopulation of resistant cells with either high SOX10 and high MITF expression or low SOX10 and low MITF expression may exist in the same tumor, rendering difficult to completely eliminate the tumor with one targeted treatment.

## Conclusion

In conclusion, stem cell-based models of human NCs represent a concrete alternative for probing melanoma progression. Reactivation of developmental signaling during the advancement of melanoma has allowed concentrating the investigations on specific genes. Nevertheless these models will allow identifying new targets which were not tested in a melanoma setting so far. Several NC differentiation methods are nowadays available but few of them lead to a pure cell population and therefore, more NC markers are still needed for better characterization. The availability of melanocyte early progenitors will greatly help the discovery of new candidate genes, targeting of which could improve current melanoma treatments or the prediction of melanoma cell response to a particular drug. MITF or SOX10 have been extensively investigated and allowed a better comprehension of resistance to cancer therapies, underlying the importance of lineage specific signaling in our understanding of how melanoma still overcomes current treatments in the clinic.

## Author Contributions

LL conceived, designed the presented idea, and wrote the manuscript. JU provided financial support and revised the written manuscript.

## Conflict of Interest Statement

The authors declare that the research was conducted in the absence of any commercial or financial relationships that could be construed as a potential conflict of interest.
